# Current situation and influencing factors of Chinese children’s diagnosis delay in autism

**DOI:** 10.1186/s11689-025-09636-2

**Published:** 2025-08-12

**Authors:** Feng-Lei Zhu, Yue Ji, Lu Wang, Min Xu, Xiao-Bing Zou

**Affiliations:** Child Developmental and Behavioral Center, the Third Affiliated Hospital of Sun Yat- sen University, Guangzhou, China

**Keywords:** Autism, Children, Diagnostic delay, Age of first concern (AOC), Age of diagnosis (AOD), The Cox proportional hazard model

## Abstract

**Background:**

Although experienced clinicians are capable of diagnosing autism in children before they reach the age of 2, the average age of diagnosis reported internationally is between 4 and 5 years, indicating a significant delay. This study aimed to determine the factors influencing the diagnostic delay time (DDT) in Chinese autistic children.

**Methods:**

We employed the Cox proportional hazard model to examine the effects of individual, family, sociodemographic, and healthcare system indicators on DDT in 480 Chinese autistic children (age range: 16.10–190.16 months; male-to-female ratio: 5.67:1) recruited from a tertiary hospital between 2021 and 2023.

**Results:**

The median DDT was 9.58 months (IQR = 15.01). Independent risk factors for delayed diagnosis included normal language competence (RR = 1.747, *p* < 0.001), non-core symptoms as first concerns (RR = 1.642, *p* = 0.013), school attendance (RR = 1.941, *p* < 0.001), irregular well-child visits (RR = 1.264, *p* = 0.028), and misdiagnosis history (RR = 0.648, *p* = 0.001).

**Conclusions:**

Diagnosis delay in Chinese autistic children is heterogeneous. Early monitoring for children with normal language skills and school-aged children, alongside improved healthcare system practices, is critical.

## Introduction

Autism is a neurodevelopmental condition characterized by social communication deficits, restricted interests, and repetitive behaviors, with a global prevalence of approximately 1–2% [1–3]. Early diagnosis before age 6 is critical, as neuroplasticity during early childhood enables interventions to improve functional outcomes, such as communication skills and adaptive behaviors [4–7]. However, delayed diagnosis remains a global challenge, with the average age of diagnosis (AOD) ranging from 4 to 5 years in high-income countries and even later in low-resource settings [8–16]. In China, where autism prevalence is estimated at 0.7%, delayed diagnosis poses a disproportionate burden due to limited diagnostic resources, cultural stigma, and delayed parental recognition of symptoms [2, 8, 17]. This delay prolongs families’ access to evidence-based interventions, exacerbating societal costs—estimated at over $2 million per individual in lifetime care—and perpetuating psychological distress linked to diagnostic uncertainty, such as caregiver burnout and social isolation [18].

### Global evidence on diagnostic delays

Diagnostic delay time (DDT), defined as the interval between initial parental concerns (age of first concern, AOC) and formal diagnosis (age of diagnosis, AOD), is influenced by complex interactions between child-level, familial, and systemic factors [19–24]. Internationally, studies report a mean DDT of 2–4 years, with AOC occurring as early as 14–24 months but AOD often delayed until school age [16, 25–30]. For example, U.S. cohorts show a mean AOD of 52 months, while U.K. studies highlight a 3.6-year gap between first healthcare contact and diagnosis [10, 27]. In low- and middle-income countries (LMICs), such as India, AOD averages 64 months, with rural populations facing longer delays due to limited specialist access [19]. Key predictors of prolonged DDT include lower socioeconomic status, co-occurring intellectual disability, female sex, and parental hesitancy to seek evaluation due to stigma or symptom misinterpretation [14–16]. Cultural factors further shape diagnostic timelines: collectivist societies may prioritize compliance over social communication differences, while Western medical systems increasingly emphasize early screening tools like the M-CHAT [31–33].

### Gaps in Chinese context

Despite China’s vast autism population, research on DDT remains sparse. Existing studies report an AOD of 3–5 years, with rural families experiencing delays 1.5 longer than urban counterparts [19, 34]. However, these findings lack granularity—critical factors such as regional healthcare disparities, the one-child policy’s impact on parental vigilance, and culturally specific symptom reporting (e.g., emphasizing “quietness” over social reciprocity deficits) remain underexplored [17, 32]. Furthermore, China’s reliance on physician-led (vs. parent-reported) symptom identification and limited autism-specific training for pediatricians may uniquely prolong DDT compared to Western systems [8, 35]. While international studies highlight systemic barriers (e.g., referral waitlists), China’s distinct healthcare infrastructure and cultural norms necessitate targeted investigations to identify modifiable drivers of delay.

### Research aims

This study addresses two unresolved questions: [1] What are the characteristics of DDT in Chinese children with autism, including AOC and AOD [2]? Which child-, family-, and system-level factors most strongly predict prolonged DDT in this population? We hypothesize that: normal language and intelligence quotient (IQ), lower parental education and knowledge of autism, attending school, comorbidity and misdiagnosis experience will correlate with longer DDT.

By elucidating these dynamics, our findings aim to inform culturally adapted strategies to reduce diagnostic delays and improve early intervention access in China.

## Materials and methods

### Study design and participants

This retrospective single-center observational study aimed to investigate factors influencing the DDT of autism in Chinese children. The study recruited 480 children diagnosed with autism at the Child Developmental and Behavioral Center of Sun Yat-sen University’s Third Affiliated Hospital between January 2021 and December 2023. Participants were aged 16.10 to 190.16 months (median age: 57.36 months, IQR = 38.70), with a male-to-female ratio of 5.67:1 (85.0% male, 15.0% female). Primary caregivers (75.6% parents) provided data through an online questionnaire.

### Inclusion and exclusion criteria

Inclusion criteria were: [1] children with a clinical autism diagnosis based on the *Diagnostic and Statistical Manual of Mental Disorders*,* Fifth Edition* (DSM-5) [3]; [2] informed consent obtained from legal guardians; and [3] completion of the online questionnaire by caregivers prior to December 2023.

Exclusion criteria included: [1] children without a primary autism diagnosis or suspected autism; [2] comorbid visual or hearing impairments; [3] presence of catatonia; or [4] questionnaires with logical inconsistencies (e.g., implausible age ranges).

### Measures

Data were collected via an online questionnaire (Wenjuanxing platform, https://www.wjx.cn) during clinical visits. The questionnaire comprised 33 items assessing clinical characteristics, sociodemographic, and healthcare processes. It was adapted from validated autism screening tools (e.g., M-CHAT-R/F [36]) and modified for cultural relevance. The questionnaire captured: Quantitative data: Age of first parental concern (AOC) and age of autism diagnosis (AOD), recorded to the nearest month. Qualitative data: clinical characteristics (e.g., language competence, comorbidities), familial antecedents (e.g., caregiver education level), sociodemographic factors (e.g., household income), and healthcare system indicators (e.g., regularity of well-child visits). DDT was calculated as the interval between AOD and AOC:

$$\mathrm{DDT}\;=\;{\mathrm T}_{\mathrm{AOD}}-{\mathrm T}_{\mathrm{AOC}}\\$$ 

Categorical variables (e.g., language competence, school attendance) were coded numerically for analysis (Table 1). Intelligence was estimated by caregivers’ subjective reports (e.g., “normal,” “marginal,” “deficient”), as standardized IQ tests were not administered. Language competence was categorized as “normal” if caregivers reported age-appropriate milestones. The internal consistency (Cronbach’s α = 0.82) and test-retest reliability (*r* = 0.78) were established in a pilot study.

**Table 1:** Main survey indicators and specific classification of each variable

Variables	Percentage (%)/Value (Mean ± SD) (*n* = 480)
1.Sex*	Male (85.0); Female (15.0)
2.Gestational age at birth	Full-term infants (89.4); Premature infants (10.6)
3.Personality types	Introverted (21.5); Intermediate (49.4); Extroverted (29.1)
4.Caregiver-reported severity of autism	Mild (47.9); Moderate (23.1); Severe (5.4); NA (23.6)
5.Language competence*	Normal (31.3); Abnormal (68.7)
6.Intelligence *	Normal and above (60.4); Marginal (19.2); Mental deficiency (20.4)
7.First concern symptom	Core defect of autism (87.1); Not autism core defect (12.9)
8.Comorbidity*	No comorbidity (11.7); At least one comorbidity (88.3)
9.Birth order	1 (62.5); 2 (32.1); 3 and above (5.4)
10.School attendance status	Attended school (50.8); Not yet in school (49.2)
11.Ethnic group	Han nationality (94.2); Ethnic minorities (5.8)
12.Diagnostic process satisfaction	Dissatisfied (9.0); Be satisfied (91.0)
13.Urbanization degree of residence	Rural areas (6.5); Towns (19.2); Cities (74.3)
14.Household annual income (ten thousand yuan)	Below 5 (5.4); 5 ~ 10 (16.0); 10 ~ 15 (15.8); 15 ~ 20 (16.3); 20 ~ 25 (9.0); 25 ~ 30 (7.5); 30 ~ 35 (5.4); 35 ~ 40 (5.4); 40 ~ 45 (3.7); 45 ~ 50 (4.0); Above 50 (11.5)
15.Number of main family members	2 (5.2); 3 (19.4); 4 (28.3); 5 (30.2); 6 and above (16.9)
16.Relationship between family members	Good (73.4); Average (23.3); Poor (3.3)
17.Family common language category	Monolingual (32.7); Bilingual or Multilingual (67.3)
18.Living with grandparents or not	Living with grandparents (68.1); Not living with grandparents (31.9)
19.Primary caregiver types	Parents (75.6); Grandparents (19.6); Others (4.8)
20.Age of primary caregiver	41.15 **±** 11.12 years
21.Language types of primary caregivers	Mandarin (72.9); Dialects (23.1); Others (4.0)
22.First noticed or concerned person	Parents (62.5); Grandparents (7.9); Others (29.6)
23.Primary caregiver educational level	Primary education (5.6); Junior school (16.3); Senior high school (10.8); College degree (21.0); Bachelor’s degree (31.9); Master’s degree (10.2); Doctoral degree (1.5); NA (2.7)
24.Paternal educational level	Primary education (0.4); Junior school (9.2); Senior high school (10.4); College degree (20.4); Bachelor’s degree (44.3); Master’s degree (11.5); Doctoral degree (3.8)
25.Maternal educational level	Primary education (0.2); Junior school (9.4); Senior high school (6.7); College degree (26.0); Bachelor’s degree (42.3); Master’s degree (13.5); Doctoral degree (1.9)
26.Paternal age at birth	31.29 ± 5.25 years
27.Maternal age at birth	29.40 ± 4.52 years
28.Parental relationship	Good (77.5); Average (17.7); Poor (4.8)
29.Familiarity with developmental milestones	Unfamiliar (14.6); Partial understanding (55.8); Familiar (29.6)
30.Knowledge of autism	Unfamiliar (75.8); Familiar (24.2)
31.Well-child care visits	Irregular participation (37.9); Regular participation (62.1)
32.Title of the initial doctor	Resident (2.1); Attending physician (22.7); Deputy chief physician (32.3); Chief physician (42.9)
33.Misdiagnosis experience	No misdiagnosis experience (78.5); Had misdiagnosis experience (21.5)

*NA* Not applicable or unclear

*Sex: a kind of biological or physiological attribute

*Language competence: evaluated based on whether their language development aligns with their physiological age and if they have reached developmental milestones (this study did not use professional tools to evaluate specific language development ages)

*Intelligence: assessed based on empirical estimation of caregivers (this study did not use standard intelligence test tools to evaluate subject’s intelligence level)

*Comorbidity: include attention deficit hyperactivity disorder, intellectual disability, tic disorder, etc

### Statistical analysis

IBM SPSS (version 20.0) was used for statistical analysis, and GraphPad Prism (version 9.5) was used for data visualization. Continuous variables with normal distributions were summarized as mean ± standard deviation (SD), while non-normal data were reported as median (interquartile range, IQR). The Cox proportional hazards regression model, a statistical method widely used to examine the relationship between covariates and the time until a specific event occurs, was employed to analyze factors influencing DDT. In this study, the endpoint was set at the age of 6 years, as this marks a critical developmental and educational transition in China. Early intervention before age 6 is strongly associated with improved outcomes for children with autism. In survival analysis terms, the endpoint defines the termination of follow-up: children diagnosed before age 6 were treated as complete observations, while those undiagnosed by 6 years were right-censored. Survival time was defined as the DDT, calculated as the interval (in months) between the age of first concern and the age of diagnosis. This design ensures alignment with both clinical priorities (early intervention) and methodological rigor in handling censored data. The variable screening method was: Forward Conditional. Key steps included:

#### Univariate analysis

All 33 variables (Table 1) were individually tested to identify potential predictors of DDT (*p* < 0.05).

#### Multivariate analysis

Variables significant in univariate analysis (e.g., language competence, school attendance) were included in a joint model to adjust for confounding effects and identify independent risk factors. This step isolates the independent effects of each predictor by accounting for interactions between variables. Statistical significance was set at *p* < 0.05.

## Results

### Descriptive results

A total of 480 children with autism (male-to-female ratio: 5.67:1; median age: 57.36 months, IQR = 38.70) were recruited from 27 provinces in China. Key characteristics of the cohort are summarized in Table 1.

**Fig. 1:**
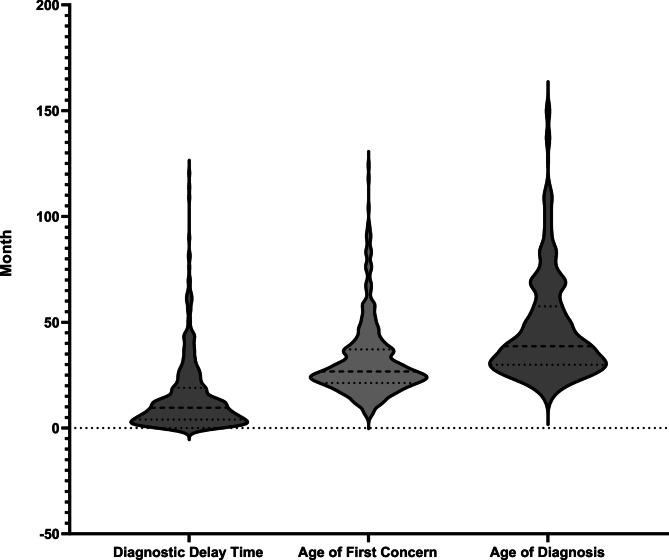
The Violin Plot of diagnostic delay time, age of first concern and age of diagnosis of children with autism

1. Primary caregivers: Parents (75.6%) were most likely to first notice developmental concerns.
2. Initial concerns: 87.1% of caregivers reported symptoms related to autistic core symptom (e.g., social communication difficulties).
3. Diagnostic outcomes: 86.5% of children (*n* = 415) were diagnosed before age 6. Median DDT was 9.58 months (IQR = 15.01), with AOC and AOD medians at 26.69 months (IQR = 15.89) and 38.65 (IQR = 27.55) months, respectively (Fig. 1; Table 2). However, 25% of these children experienced delays exceeding 24.59 months. The remaining 65 children (13.5%) received diagnoses after age 6, with a median DDT of 43.2 months (IQR = 28.7). Further analysis revealed that this subgroup had higher rates of normal language competence (42.3% vs. 29.1%, *p* = 0.02) and irregular well-child visits (58.5% vs. 34.9%, *p* < 0.001) compared to those diagnosed earlier.

**Table 2:** Key characteristics of age of first concern, age of diagnosis and diagnostic delay time

Variable	Median (IQR)	Range (months)
Age of first concern	26.69 (15.89)	6.14-124.25
Age of diagnosis	38.65 (27.55)	12.78-152.61
Diagnostic delay time	9.58 (15.01)	0.53-120.44

### Factors influencing diagnostic delay

#### Univariate Cox regression analysis

**Fig. 2:**
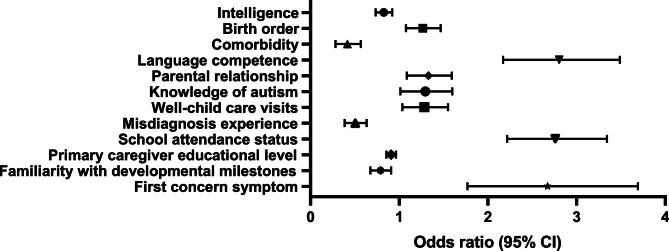
Forest Plot of univariate Cox regression results

Univariate analysis identified 12 variables significantly associated with DDT (*p* < 0.05), including: normal language competence (*RR* = 2.75), attended school (*RR* = 2.72), normal intelligence (*RR* = 0.82), first born (*RR* = 1.26), familiar with developmental milestones (*RR* = 0.78), familiar with knowledge of autism (*RR* = 1.27), misdiagnosis history (*RR* = 0.49), with comorbidities (*RR* = 0.40), good parental relationship (*RR* = 1.32), regular Well-child visits (*RR* = 1.27), and first concern symptom (*RR* = 2.56). Full results are visualized in Fig. 2 and detailed in Table 3.

**Table 3:** Hazard ratios (RR) and 95% confidence intervals for 12 significant variables

Variable	RR (95% CI)	*P* Value
Intelligence	0.822 (0.734–0.921)	< 0.001
Birth order	1.257(1.076–1.468)	0.004
Comorbidity	0.4 (0.283–0.567)	< 0.001
Language competence	2.752 (2.171–3.488)	< 0.001
Parental relationship	1.315 (1.085–1.592)	0.005
Knowledge of autism	1.273 (1.014–1.598)	0.038
Well-child care visits	1.267 (1.035–1.551)	0.022
Misdiagnosis experience	0.494 (0.385–0.634)	< 0.001
School attendance status	2.722 (2.217–3.342)	< 0.001
Primary caregiver educational level	0.908 (0.855–0.965)	0.002
Familiarity with developmental milestones	0.783 (0.675–0.909)	0.001
First concern symptom	2.556 (1.77–3.693)	< 0.001

#### Multivariate Cox regression analysis

**Fig. 3:**
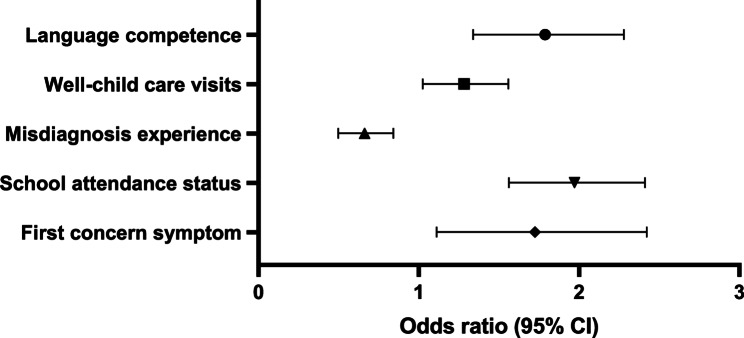
Forest Plot of multivariate Cox regression results

The multivariate model incorporated all 12 variables from univariate analysis. After adjusting for confounders, five independent predictors of DDT were identified (Fig. 3; Table 4). Risk factors: normal language competence (RR = 1.75, 95% CI: 1.34–2.28, *p* < 0.001), attended school (RR = 1.94, 95% CI: 1.56–2.41, *p* < 0.001) and misdiagnosis history (RR = 0.65, 95% CI: 0.50–0.84, *p* = 0.001). Protective factors: initial concern about core autism symptoms (RR = 1.64, 95% CI: 1.11–2.42, *p* = 0.013) and regular well-child visits (RR = 1.26, 95% CI: 1.03–1.56, *p* = 0.028). Variables such as birth order, comorbidity, and parental relationship lost significance in the multivariate model, suggesting their effects were mediated by other factors.

**Table 4:** Hazard ratios (RR) and 95% confidence intervals for 5 significant variables

Variable	RR (95% CI)	*P* Value
Language competence	1.747 (1.339–2.280)	< 0.001
Well-child care visits	1.264 (1.025–1.559)	0.028
Misdiagnosis experience	0.648 (0.498–0.842)	0.001
School attendance status	1.941 (1.563–2.411)	< 0.001
First concern symptom	1.642 (1.112–2.423)	0.013

## Discussion

Early diagnosis of autism is pivotal for accessing interventions, yet significant delays persist globally. This study identified five factors prolonging diagnostic delay in Chinese children: typical language development, non-core initial concerns, school attendance, irregular well-child visits, and prior misdiagnosis. Below, we contextualize these findings, address limitations, and propose implications for practice.

### Language competence and diagnostic delay

Our findings indicate that children with typical language abilities experience longer diagnostic delays. This aligns with prior studies showing that caregivers and clinicians often prioritize language delays as primary concerns [20, 37]. For example, parents of children with limited verbal communication are more likely to seek early evaluations, whereas those with fluent speech may overlook social or behavioral differences [23]. However, our findings emphasize that normal language skills may mask social communication deficits, leading caregivers and clinicians to overlook autism-specific traits. This underscores the need for broad developmental screening beyond language milestones, particularly in cultures where social communication differences are less stigmatized. Consequently, healthcare providers should emphasize comprehensive developmental monitoring even in children with age-appropriate language skills.

### Symptom specificity and diagnostic efficiency

Children whose first observed symptoms aligned with core features of autism (e.g., social communication challenges) received faster diagnoses. This is consistent with studies highlighting that atypical motor or sensory profiles—common in females—often delay diagnosis due to misalignment with traditional diagnostic criteria [38, 39]. For instance, girls presenting with motor delays may be misclassified until social demands increase in school settings. This mirrors clinical observations where “red flags” (e.g., lack of joint attention) prompt immediate referrals [30]. Conversely, atypical presentations complicate differential diagnosis, delaying specialist evaluations. These findings advocate for training primary care providers to recognize diverse autism phenotypes, especially in underrepresented groups.

### Misdiagnosis and systemic barriers

Children with prior misdiagnoses (e.g., attention deficit hyperactivity disorder, ADHD or intellectual disability) faced prolonged delays, likely due to overlapping symptoms and limited specialist access in China [35]. A recent multinational study found that 66.5% of autistic individuals received at least one incorrect psychiatric diagnosis before autism identification [40]. This highlights systemic challenges in differentiating autism from comorbid conditions, particularly in regions with scarce pediatric mental health resources.

### Role of Well-Child visits

Irregular participation in developmental screenings significantly extended delays. Regular checkups enable primary care providers to detect subtle signs (e.g., lack of joint attention) and refer families to specialists [8]. In contrast, caregivers relying on informal observations may misinterpret developmental differences as transient issues. Strengthening routine screening programs in rural and urban clinics could mitigate this gap.

### School attendance and missed opportunities

Children already attending school experienced longer delays, likely because educators and parents prioritized academic performance over social challenges. Studies in low-resource settings similarly report that school-based screenings significantly reduce diagnostic delays [10]. Proactive screening in educational settings—particularly for older children—could address this systemic oversight.

### Negative findings and heterogeneity

Contrary to prior research, autism severity and IQ did not predict delays in our study. This discrepancy may stem from caregiver-reported (vs. clinically assessed) severity/IQ data, which lack precision [41]. Additionally, while lower IQ is often linked to earlier diagnosis in Western cohorts [38], cultural differences in symptom recognition may explain this divergence. These “negative” results highlight the heterogeneity of diagnostic pathways and the need for culturally adapted tools.

### Implications for early vs. accurate diagnosis

While early diagnosis is critical for intervention, our findings caution against prioritizing speed over accuracy. Pressures to diagnose quickly may increase misdiagnosis risks, particularly in children with subtle presentations or comorbidities. For example, ADHD-like hyperactivity in autistic children may lead to premature diagnostic conclusions without comprehensive evaluations [22]. Clinicians must balance timely identification with rigorous assessments to avoid diagnostic overshadowing.

### The subgroup of children diagnosed after the age of 6

While the majority of children in our cohort were diagnosed promptly, the prolonged delays in 25% of cases and the 13.5% diagnosed after age 6 underscore systemic challenges. Late-diagnosed children often exhibited subtler symptoms (e.g., preserved language skills) or faced barriers such as limited access to specialized care in rural regions. These findings align with global reports linking delayed diagnosis to atypical phenotypes and healthcare inequities [25, 31]. To address this, we recommend expanding school-based screening programs and training primary care providers to recognize autism in older children with non-core symptoms.

### Limitations and future directions

This study has five key limitations:

1. Selection Bias: Our sample excluded undiagnosed children, potentially underestimating delays in severe cases.
2. Recall Bias: Retrospective caregiver reports may inaccurately reflect developmental timelines.
3. Age Threshold: The upper age limit of 15 years was chosen to align with China’s compulsory education system, where developmental concerns often emerge during school entry. However, this may exclude adolescents with late-onset symptoms.
4. Regional Bias: Participants were predominantly from South China, limiting generalizability.
5. COVID-19 Impact: Pandemic-related healthcare disruptions may have exacerbated delays [42, 43].

Future research should:

1. Use prospective cohorts to track undiagnosed children.
2. Incorporate standardized clinical assessments (e.g., ADOS-2) to validate caregiver-reported data.
3. Explore cultural perceptions of developmental milestones influencing help-seeking behaviors.

## Conclusions

Diagnostic delays in Chinese autistic children are influenced by language abilities, initial symptom profiles, school attendance, healthcare access, and prior misdiagnoses. To address these, we recommend: [1] Enhanced screening protocols in schools and primary care [2]. Caregiver education on non-language-related autism features [3], Specialist training to differentiate autism from comorbidities. By addressing these gaps, China can reduce diagnostic delays while upholding diagnostic accuracy, ultimately improving outcomes for autistic children and their families.

## Data Availability

Data is provided within the manuscript.
